# Inhibition of vitamin D analog eldecalcitol on hepatoma *in vitro* and *in vivo*

**DOI:** 10.1515/med-2020-0137

**Published:** 2020-07-13

**Authors:** Limin Ye, Liyi Zhu, Jinglin Wang, Fei Li

**Affiliations:** Department of Gastroenterology, People’s Hospital of Guizhou Province, No. 83, Zhongshan East Road, Nanming District, Guiyang, Guizhou 550002, China

**Keywords:** hepatoma, eldecalcitol, vitamin D analog, E-cadherin, Akt

## Abstract

Hepatoma is a serious liver cancer with high morbidity and mortality. Eldecalcitol (ED-71), a vitamin D analog, is extensively used as anti-cancer agent *in vitro*. Hepatocellular carcinoma cell, SMMC-7721 cell lines were used in this study. Transwell assay, cell apoptosis and cell cycle detection assays were investigated after treatment with ED-71 and phosphate buffered saline (PBS) as control. Sizes of tumors were measured after ED-71 treatment in a mouse model. E-cadherin and Akt gene expressions were detected by real-time PCR (RT-PCR). The results showed that cell invasion and migration were decreased markedly after ED-71 treatment compared to control group. Cell cycle detection showed that the G2 stage was 13.18% and total S-stage was 41.16% in the ED-71 group and G2 stage: 22.88%, total S-stage: 27.34% in the control group. Cell apoptosis rate was promoted in the ED-71 group. Size of the tumors reduced more after the ED-71 treatment than the PBS treatment in mice. ED-71 markedly inhibited the expression of Akt and E-cadherin, either detected by immunohistochemistry or RT-PCR. ED-71 treatment can inhibit the hepatoma agent proliferation by increasing the E-cadherin expression and decreasing Akt expression. Therefore, these findings provide novel evidence that ED-71 can be used as an anti-hepatoma agent.

## 1 Introduction

Hepatocellular carcinoma (HCC) is one of the most common liver cancers globally causing high risk of death in spite of advanced medical diagnosis by liver transplantation or ablation treatment, new molecular technologies [1,2]. The key technical challenge is how to identify the higher risk stage of malignant transformation patients [3]. Although histopathology diagnostics combined with advances in different forms of surgical or chemotherapy therapy have improved the results for tumor patients to a large extent [4,5], there are still no effective drugs used to inhibit tumor cell growth clinically [6]. The only systemic therapy drug for HCC is sorafenib, which is an oral multikinase inhibitor only for patients with inoperable or advanced HCC [7]. Therefore, there should be many challenges for finding the drugs curing the HCC.

Eldecalcitol, (1α, 25-dihydroxy-2β-[3-hydroxypropyloxy] vitamin D3), also called ED-71, which is a novel analog of vitamin D, has been widely used for the treatment of osteoporosis [8]. Eldecalcitol plays an important role in regulating the metabolism of bone as well as calcium, it was particularly used for patients who were suffering from vitamin D deficiency. Clinical trials suggested that eldecalcitol possessed a strong inhibitory influence on the resorption of bone [9,10]. However, recent reports have showed that eldecalcitol can also be used for anti-cancer function of oral squamous cell carcinomas (OSCCs) *in vitro* by inhibiting the cancer cell line growth [11]. Eldecalcitol can affect the substance of Cyp24A1 expression in the cancer cells [12]. Due to the Cyp24A1 mRNA expression up-regulation and interaction with the calcitriol anti-proliferative functions [13], the calcitriol level may decrease to prevent the application of vitamin D3 for the therapy of different cancers. Therefore, eldecalcitol was used for substituting the vitamin D3 avoiding the hypercalcemia.

Meanwhile, whether eldecalcitol has an inhibiting effect on the hepatoma agents is still unknown. Herein, we explored the function of eldecalcitol on the hepatoma cell including E-cadherin and Akt gene expression change for the first time; further, the features of transwell assays, such as cell invasion and migration, cell apoptosis as well as cell cycles, were measured post ED-71 treatment. In addition, with a mouse model, the tumor cell growth was compared post eldecalcitol treatment and the tumor tissues were analyzed by immunohistochemistry experiments. Taken together, this work may offer a novel view for hepatoma therapy.

## 2 Materials and methods

### 2.1 Cell culture

HCC cell, SMMC-7721 cell line purchased from the Chinese Academy of Sciences (Beijing, China) was used in the study. All cells were cultured in the RPMI medium mixed with 10% fetal bovine serum (FBS), penicillin–streptomycin (100 IU/mL), and trypsin (100 µg/mL) in a cabinet (Thermo Scientific, USA) containing 5% CO_2_ and saturated humidity at 37°C. One milliliter of trypsin was added for digestion for 1 min, followed by adding 2 mL of complete medium to terminate the digestion. After centrifugation (1,000 rpm) for 3 min, the precipitated cells were collected, and the new cell suspension was re-suspended with complete medium to be transferred or inoculated in the required proportion.

### 2.2 Flow cytometry analysis

Flow cytometry analysis was conducted, as described previously [14,15]. Cells (8 × 10^3^) were seeded into 6-well plates and cultured for 24 h in a cabinet. After treatment with ED-71 for another 48 h, cells (1 × 10^5^/mL) were digested, and centrifuged to collect the cell pellet, and then suspended in 1 mL binding buffer containing 10 µL Annexin V-FITC (Haoxin Biotech, China) and 10 µL propidium iodide (PI) (Abcam Biotech, UK). After incubation for 10 min at room temperature in the dark, apoptosis was counted through flow cytometry. The apoptotic rate was scored by quantifying apoptosis (Annexin V-FITC + PI).

### 2.3 Cell intervention experiment

The cells were digested and counted when cell density reached 80% confluence. The cells were then transferred to 6-well plates according to the density of 8 × 10^5^/mL and cultured overnight in the incubator. On the following day, the drug was added to the 6-well plate by ED-71 (0.5 nM) treatment and PBS as control. After ED-71 was added, the cells were cultured for 48 h. Cell apoptosis and cycles were performed by the flow cytometry with the Annexin-V and PI kit.

### 2.4 Transwell assay

Transwell assay was conducted, as described previously [16]. Briefly, serum-free cell suspensions (2.5 × 10^4^ cells/mL) were made and 0.1 mL of the cell suspension was seeded to the top chamber of the transwell plates. Culture medium containing 10% FBS was added into the lower chamber. Cells were cultivated for 24 h at 37°C 5% CO_2_. Membranes were cleaned using a cotton swab, followed by fixing with 4% polyformaldehyde (10 min). After washing twice, the top chamber was stained with 0.5% crystal violet (Sigma-Aldrich; St. Louis, USA) for 15 min at room temperature. The experiments were performed in triplicate. Cells were observed and counted under Olympus CX43 light microscope (×40 magnification). Then invasive cells in three fields were counted, and the average number was calculated.

### 2.5 Wound healing assay

Cells (2 × 10^3^) were seeded in a 6-well plate and cultured at 37°C 5% CO_2_. When cell density reached 80% confluence, a strict line was conducted using a sterile 1 mL pipette tip. After 48 h incubation, migrated distance of cells was calculated, and pictures were captured. The migrated distance after 48 h in three fields was counted, and the average number was calculated.

### 2.6 Immunohistochemistry experiment

The tumor tissues were fixed in 10% formalin overnight. The tissues were embedded in optimal cutting temperature (OCT, tissue freezing medium) compound (Bai’ao Biotech, China). The embedded tissues were cross-sectioned in 12 µm thickness. After antigen repair, tissues were washed three times (3 min/time). Three percent H_2_O_2_ was used to culture tissues for 10 min at room temperature. After washing three times (3 min/time), 10% goat serum was used for blocking. The primary antibodies of Akt (Rabbit anti-Akt, ab179463, Abcam, UK) and E-cadherin (Rabbit anti-E-cadherin, ab40772, Abcam, UK) were used to culture tissues at 4°C overnight. After washing, the tissues were incubated with goat anti-rabbit IgG (ab205718, Abcam, UK) for 1 h. Color development reagent, DAB reagent, was used to incubate with tissues, and photographed, and analyzed using an Olympus BX41 microscope (Tokyo, Japan).

### 2.7 RNA isolation and real-time PCR (RT-PCR)

RNA isolation and real-time PCR were performed, as described previously [17]. RNA isolation: Cells were subjected to extract total RNA using TRIzol Reagent (Invitrogen, Life Technologies, USA), according to the manufacturer’s protocol. To remove any residual DNA, RNase-free DNase I was included to treat the aqueous phase at 37°C for 20 min.

RT-PCR: 1 µg of RNA was added to the reverse transcription system of oligo primer (1 µL), reverse transcriptase mix (10 µL), and RNase-free water with the PrimeScriptTM RT Reagent Kit, according to manufacturer’s instruction. The RT-PCR system includes cDNA, the primers, 2× plus SYBR real-time mixture, ddH_2_O, ROXI of the ChamQTM SYBR^®^ qPCR Master Mix (Vazyme Biotech Co., Ltd, China), and DEPC-treated water (Mellon Biological Services, USA). The GAPDH forward primer: ATGGGGAAGGTGAAGGTCG, reverse primer: TCGGGGTCATTGATGGCAACAATA; the E-cadherin forward primer: GGCTGGACCGAGAGAGTTTC, reverse primer: TCAAAATCCAAGCCCGTGGT; the Akt forward primer: GGCGGCAGGACCGAG, reverse primer: CGCCTGCTCCCGTCTTC, were synthesized by Shengya Biosynthesis Company, Fuzhou, China. The PCR steps were initial denaturation 95°C for 2 min, 40 cycles of 95°C for 15 s, and 60°C for 60 s. Data were analyzed by relative quantification expression normalized to glyceraldehyde-3-phosphate dehydrogenase (GAPDH). Target gene mRNA expression levels between the treatment group and the control group were calculated using the 2^−ΔΔCt^ method.

### 2.8 Establishment of HCC transplantation model in mice

Nude mice (C57BL/6, 6 weeks old) were purchased from Beijing Vital River Laboratory Animal Technology (Beijing, China). Information of animal experiment is shown in Table 1. The animal experiments were approved by the People’s Hospital of Guizhou Province, and animal experiments described in this study were performed in accordance with established procedures, as defined by the Guideline on the Humane Treatment of Laboratory Animals stipulated by the Ministry of Science and Technology of the People’s Republic of China [18]. SMMC-7721 cells (5 × 10^6^, 0.1 mL) were subcutaneously given into the stomach of mice. When the tumor grew to around 7 mm, mice were randomly divided into two groups, ED-71 treatment (0.2 µg/kg) group and PBS treatment as control, each group included six mice. After 21 days, mice were sacrificed and tumors were harvested. The size and weight of the tumor were measured.

**Table 1:** Information of animal experiment

Group	Control	ED-71
Animal number	6	6
Gender	Male	Male
Baseline weight (g)	17.9 ± 1.3	17.5 ± 1.0

### 2.9 Statistical analysis

The data were showed as mean values with standard deviation (SDs). Statistical significance was determined by the Student’s *t*-test, and a *P* value < 0.05 was considered statistically significant. All the results were obtained in at least three independent repeated experiments.

## 3 Results

### 3.1 ED-71 inhibited the migration and invasion of hepatoma cells

**Figure 1:**
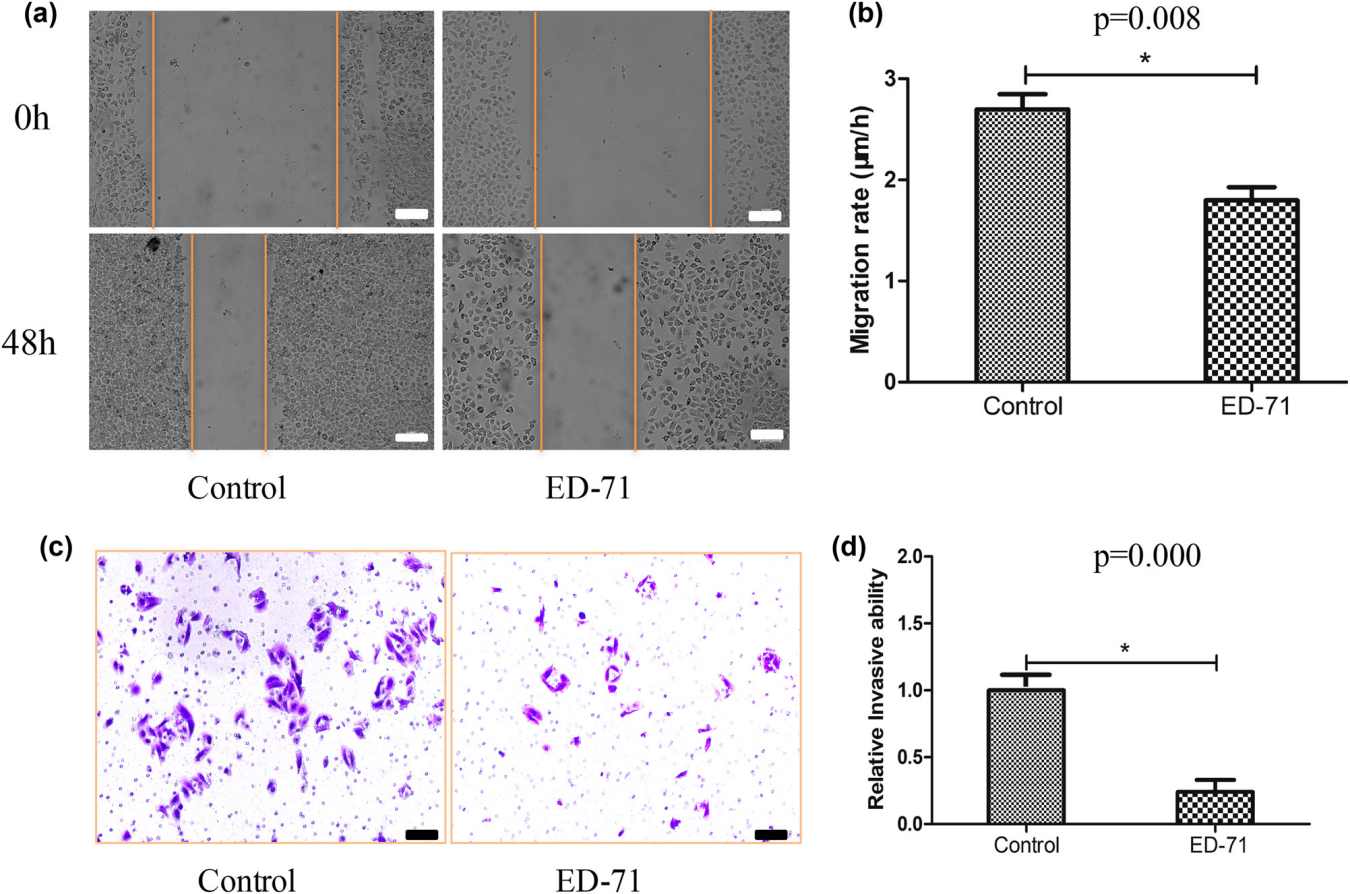
ED-71 (0.5 nM) markedly inhibited the migration and invasion of liver cancer cells. (a) Representative pictures of cell migration after treatment with ED-71 (scale bar: 150 µm); (b) quantification analysis of cell migration after treatment with ED-71; (c) representative pictures of cell invasion after treatment with ED-71 (scale bar: 30 µm); (d) quantification analysis of cell invasion after treatment with ED-71. *P < 0.05 compared with the control group.

Cell migration and invasion have been believed to be closely related to tumor metastasis. We investigated the influence of ED-71 (0.5 nM) on the migration and invasion of SMMC-7721 using wound healing and transwell assays, respectively. We found that after treatment with ED-71, the migration rate was decreased significantly (Figure 1a and b). Meanwhile, the invasion of cells was also suppressed significantly by ED-71 (Figure 1c and d).

### 3.2 ED-71 markedly increased the ratio of hepatoma cells of S stage and decreased G2 of the cell cycle

**Figure 2:**
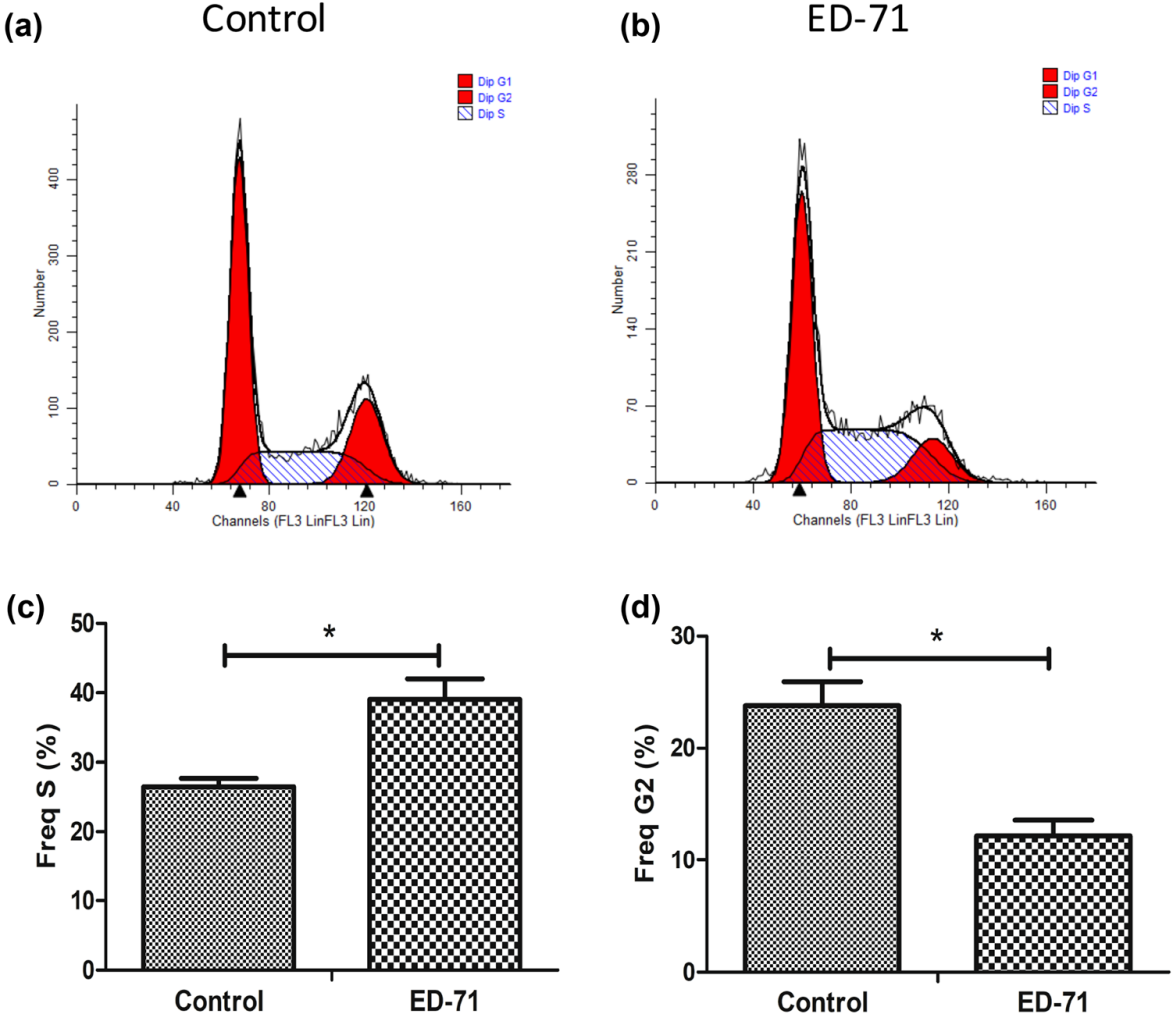
ED-71 (0.5 nM) markedly increased the percentage of liver cancer cells in S stage and decreased the percentage in G2 stage of the cell cycle. (a) Representative picture of cell cycle was measured without ED-71 treatment; (b) representative picture of cell cycle was measured after treatment with ED-71; (c) quantification analysis of cells in S stage after treatment with ED-71; (d) quantification analysis of cells in G2 stage after treatment with ED-71. *P < 0.05 compared with the control group.

To explore how ED-17 affects the cell cycle and apoptosis of the hepatoma cells, we detected the cell cycle post the drug treatments with flow cytometer. The results demonstrated that G2 phase was 13.18% and total S-phase was 41.16% in the ED-71 group and G2 phase: 22.88%, total S-phase: 27.34% in the control group (Figure 2). Meanwhile, the percentage of the cells in S stage increased post ED-71 (0.5 nM) treatment but the G2 phase opposite. The results indicated that ED-17 plays a vital role in the cell division, proliferation, and survival.

### 3.3 ED-71 notably induced cell apoptosis rate and inhibited tumor growth *in vivo*

**Figure 3:**
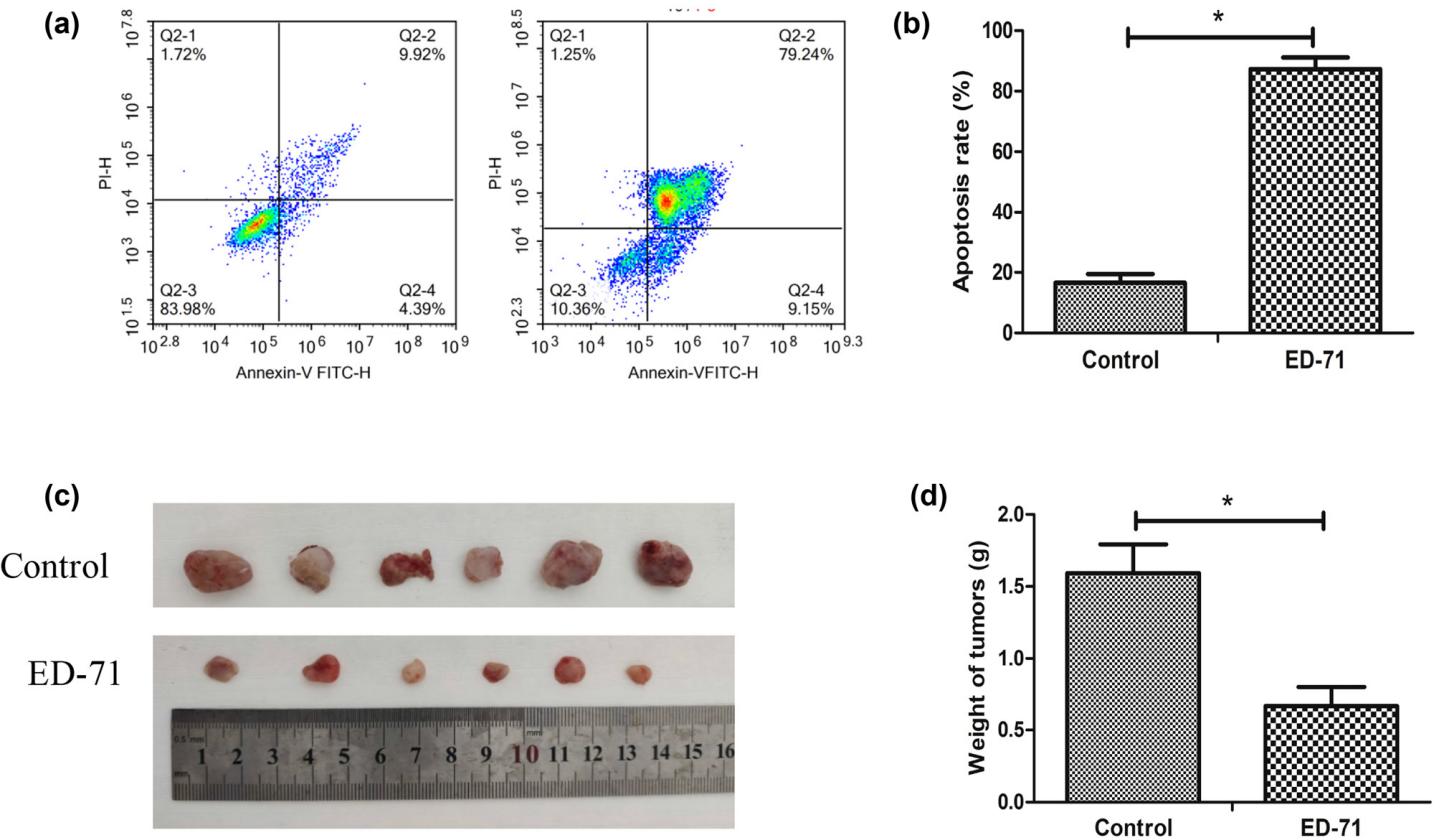
ED-71 induced cell apoptosis of human liver cancer cells using flow cytometry analysis and markedly inhibited the growth of tumor in mice. (a) Cell apoptosis was measured after treatment with ED-71 (0.5 nM); (b) quantification analysis of cell apoptosis after treatment with ED-71; (c) representative pictures of tumor after treatment with ED-71 (0.2 µg/kg); (d) measurement of tumor weight. *P < 0.05 compared with the control group.

To investigate the function of the ED-71 on HCC, the transplanted tumor model was established. Each group has six mice, which were given a gavage of distilled ED-71 in the treatment group and the same volume of PBS in the control group. The mice were dissected for collecting the tumor tissues 21 days after treatment. The tumor size of the ED-71 treatment group obviously narrowed compared with the tumors in PBS control group (Figure 3b and d). Through the figures of the tumors, we can come to the conclusion that the ED-71 indeed did suppress the growth of the tumor cells *in vivo*. And in addition, study *in vitro* showed that ED-71 increased cell apoptosis rate which is 90% in the treatment group more than that of 14.5% in the control group (Figure 3a and c).

### 3.4 ED-71 increased the E-cadherin expression and decreased the Akt expression

**Figure 4:**
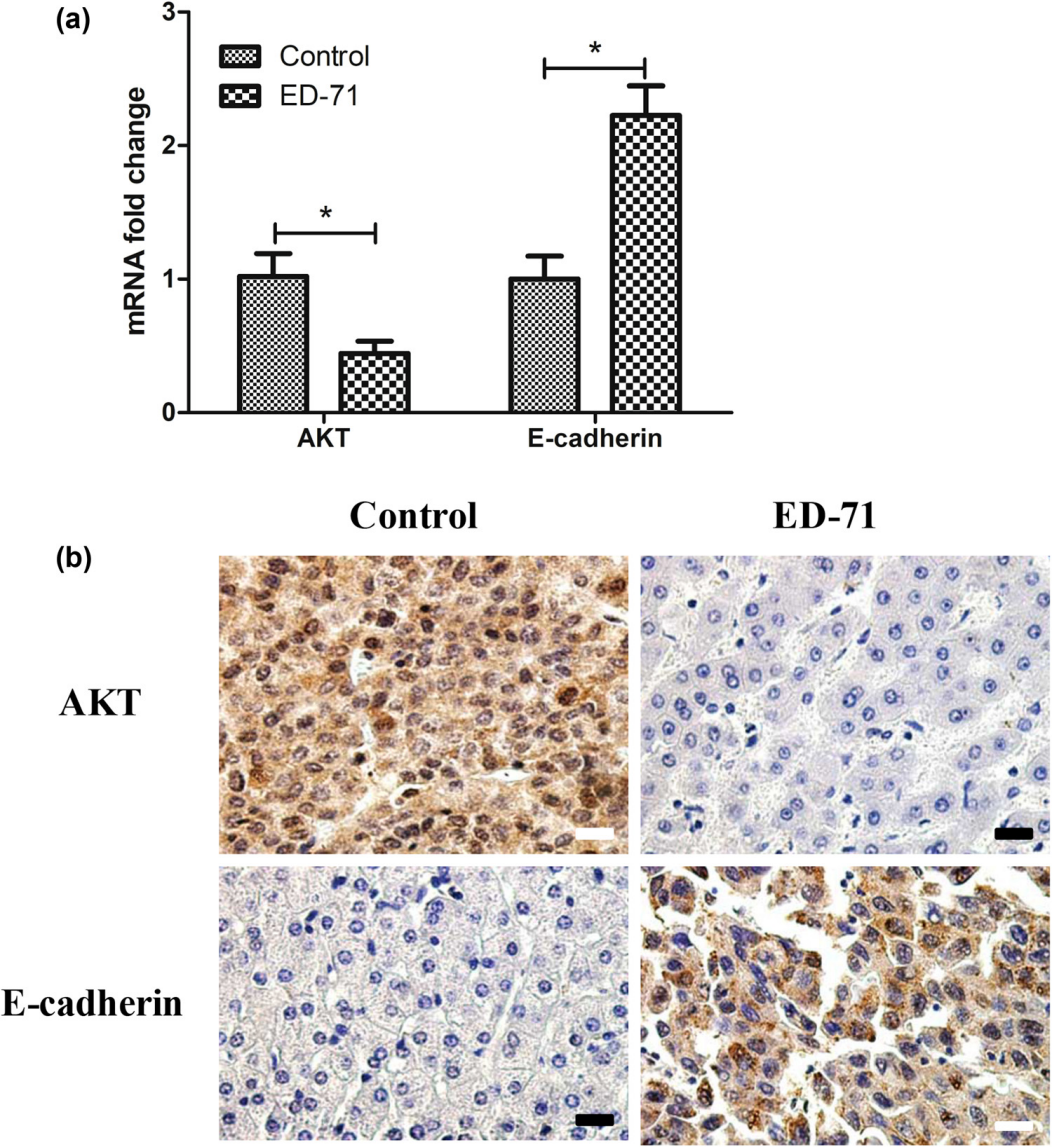
ED-71 (0.2 µg/kg) markedly inhibited the expression of Akt and E-cadherin. (a) ED-71 increased the E-cadherin mRNA expression and decreased the Akt expression; (b) expression of Akt (Rabbit anti-Akt) and E-cadherin (Rabbit anti-E-cadherin) was measured by immunohistochemistry staining after treatment with ED-71 (scale bar: 50 µm). *P < 0.05 compared with the control group.

E-cadherin was related to the absorption of calcium for the cells, so we detected the mRNA expression post ED-71 treatment. The RT-PCR results demonstrated that E-cadherin expression increased more than 2-fold in the treatment group than in the blank group (Figure 4a). Consequently, the ED-71 increased the E-cadherin mRNA expression for inhibiting the tumor cell growth. However, some anti-cancer drugs can activate the Akt/mTOR signaling pathways. Hence, we also explored the Akt expression after the ED-71 treatment. Results demonstrated that the mRNA expression decreased in the treatment group than in the blank group. Results of immunohistochemistry of tumors indicated that ED-71 induced the E-cadherin expression but suppressed the Akt expression of the tumor cells for growth (Figure 4b).

## 4 Discussion

1. Many previous preclinical researches indicated that analogs of the vitamin D such as calcitriol have the potential as anti-cancer agents for human health [19,20,21]. However, these agents may cause the secondary side effect including hyperparathyroidism [22]. There is still a long way to find a relatively perfect drug for the cancer therapy. As a matter of fact, the analog vitamin D [1, 25(OH)_2_D_3_] is found to be a key player in the treatment of hyperparathyroidism [23]. Reports showed that the vitamin D analog 1α,25(OH)_2_D_3_ may be effective for the treatment of oral squamous cell carcinoma by inhibiting the activity of the NF-κB [24]. While ED-71 is quite a new drug of the vitamin D analog used majorly for anti-osteoporosis [25,26], instead of anti-cancer. Recent research showed the anti-cancer function of the drug on the OSCC [27], giving a new sight that ED-71 is a potential anti-cancer agent for OSCC [28,29]. Actually, HCC also requires the effective anti-cancer drugs. Therefore, this is a novel study to first investigate the ED-71 effect on the HCC. We found that ED-71 could decrease proliferation of HCC cells obviously *in vitro* as well as inhibiting the growth *in vivo* of the mouse model.
2. The anti-cancer drug used in clinic may be verified for the influence on the cell division *in vitro* of the cancers [30]. Danusertib induces cell cycle arrest in G2/M phase in HCC HepG2 cells [31]. Curine induces cell cycle arrest and cell death in HCC cells in a p53-independent way [32]. α-Pinene could inhibit miR221 expression leading to G2/M-phase arrest, so as to suppress hepatoma tumor progression [33]. Here we found that the percentage of the cells in S stage increased post ED-71 treatment but the G2 phase decreased, which is consistent with the above drugs causing the G2 phase. And in other cancers such as lung cancer, the percentage of lung cancer cells in S and G2 stages was increased markedly by Chinese herbal formulas Miao–Yi–Ai–Tang [34]. These results indicated the potential function of the ED-71 anti-cancer agent. More importantly, we discovered that ED-71 increased the cell apoptosis ratio than the control group, which affirmed the inhibition effect of the ED-71, although the detailed pathways and mechanism still need to be explored.
3. Results of the *in vivo* experiment of the mouse tumor model also proved the anti-cancer function that post gavage the mice with ED-71, the tumor growth was markedly reduced. Combined with the immunohistochemistry staining of the tumor tissues, less expression of the tumor was found after the ED-71 treatment. These results revealed the anti-cancer effect of the drug. As the prior research observed, the reduction in tumor size and an increase in the calcium level in the blood of mice treated with ED-71 in the OSCC [11], measuring the calcium level, also need to be strengthened in the later research to analyze the inhibition mechanism [26,35].
4. The migration and invasion of tumor cells have been proved to be closely linked with the tumor metastasis. In this study, we demonstrated that ED-71 markedly inhibited the invasion and migration of hepatoma cells, indicating that ED-71 might be a potential anti-tumor agent. The migration and invasion of tumor cells are regulated by many signaling pathways including Akt/JNK [36]. Meanwhile, the epithelial–mesenchymal transition (EMT) is a key step in the metastasis of tumor, and it is closely related to the migration and invasion of tumor cells. Therefore, the major target molecule, E-cadherin, was investigated in the present study.

E-cadherin, also known as epithelial cadherin, is applied for the diagnosis as well as prognosis of the epithelial cancers [37]. It plays an important role in suppression versus initiation or progression of various human cancers [37,38]. Furthermore, the Akt/PKB kinases can be frequently activated in human cancers including oral squamous cell carcinoma [39]. Akt is activated in many human carcinomas. Akt can induce the EMT by down-regulation of the E-cadherin expression. The Akt can simultaneously induce EMT, so as to promote enhanced motility and invasiveness of squamous cell carcinoma lines [40]. In this study, we have measured the mRNA expression of the E-cadherin and Akt post the ED-71 treatment by RT-PCR. E-cadherin expression increased more than 2-fold and Akt expression decreased about 1-fold in the treatment group than in the control group, which proved that ED-71 can inhibit the hepatoma growth by inducing the expression of E-cadherin but suppression of the Akt expression. More protein pathways should be explored with the western blotting and structure analysis afterwards.

Apart from Akt and E-cadherin, many other potential factors have been proved to be regulated by ED-71 affecting tumor cells. Previous study indicated that ED-71 could suppress oral squamous cell carcinoma by inhibiting HBp17/FGFBP-1, FGF-2, CD31, Ki-67, and Cyp24A1 [27,28]. However, if ED-71 could suppress the hepatoma cells through affecting these factors described above remains unknown. It should be an interesting study to investigate the influence of ED-71 on the expression of these factors in hepatoma cells.

Taken together with the findings above, we can draw a conclusion that the vitamin D analog eldecalcitol, ED-71, is a potential therapeutic agent for anti-hepatoma *in vitro* and *in vivo*, which will provide a novel sight for inhibiting the hepatoma cancer.
